# V3-Independent Competitive Resistance of a Dual-X4 HIV-1 to the CXCR4 Inhibitor AMD3100

**DOI:** 10.1371/journal.pone.0089515

**Published:** 2014-02-19

**Authors:** Yosuke Maeda, Hiromi Terasawa, Yusuke Nakano, Kazuaki Monde, Keisuke Yusa, Shinichi Oka, Masafumi Takiguchi, Shinji Harada

**Affiliations:** 1 Department of Medical Virology, Faculty of Life Sciences, Kumamoto University, Kumamoto, Japan; 2 Division of Biological Chemistry and Biologicals, National Institute of Health Sciences, Tokyo, Japan; 3 AIDS Clinical Center, National Center for Global Health and Medicine, Tokyo, Japan; 4 Center for AIDS Research, Kumamoto University, Kumamoto, Japan; Centro de Biología Molecular Severo Ochoa (CSIC-UAM), Spain

## Abstract

A CXCR4 inhibitor-resistant HIV-1 was isolated from a dual-X4 HIV-1 *in vitro*. The resistant variant displayed competitive resistance to the CXCR4 inhibitor AMD3100, indicating that the resistant variant had a higher affinity for CXCR4 than that of the wild-type HIV-1. Amino acid sequence analyses revealed that the resistant variant harbored amino acid substitutions in the V2, C2, and C4 regions, but no remarkable changes in the V3 loop. Site-directed mutagenesis confirmed that the changes in the C2 and C4 regions were principally involved in the reduced sensitivity to AMD3100. Furthermore, the change in the C4 region was associated with increased sensitivity to soluble CD4, and profoundly enhanced the entry efficiency of the virus. Therefore, it is likely that the resistant variant acquired the higher affinity for CD4/CXCR4 by the changes in non-V3 regions. Taken together, a CXCR4 inhibitor-resistant HIV-1 can evolve using a non-V3 pathway.

## Introduction

The entry of human immunodeficiency virus type 1 (HIV-1) is initiated by an interaction of viral envelope glycoprotein gp120 with the principal receptor CD4 and one of the coreceptors, either CCR5 or CXCR4, expressed in the target cells. HIV-1 is classified into three phenotypes based on its ability to use CCR5 (R5), CXCR4 (X4), or both (R5X4 or dual-tropic). Certain dual-tropic viruses are further classified into those that prefer CCR5 (dual-R5) or CXCR4 (dual-X4) [1], [2]. It has been shown that the coreceptor usage of HIV-1 is mainly determined by the third variable region of gp120 (V3 loop) [3], [4], [5] that is composed of ∼35 amino acids. In particular, the number and position of positively charged amino acids in the V3 loop are important for coreceptor selectivity such as the 11/25 rule. If the 11th or 25th positions of the V3 loop are positively charged, viruses will use CXCR4. Otherwise, they will use CCR5 [6]. Lack of an N-linked glycan at the 6th position of the V3 loop is also involved in CXCR4 usage [7], [8], [9]. In general, R5 viruses are predominant in the early stage of infection, whereas CXCR4-using viruses (dual-tropic and X4 viruses) emerge at the late stage of infection and are associated with disease progression in half of HIV-1-infected individuals [10], [11], [12]. It has been postulated that coreceptor inhibitors or natural ligands of CCR5 or CXCR4 might induce the coreceptor shift of HIV-1 between CCR5 and CXCR4. However, *in vitro* studies have shown that these escape variants acquired resistance using the same coreceptor. For example, MIP-1α (a natural ligand for CCR5)-induced escape variants of R5 HIV-1 and selected viruses exhibit substitutions in the V2 region and V3 loop without changing CCR5 usage of the virus [13]. CCR5 inhibitors such as maraviroc (MVC) and vicriviroc also do not change coreceptor usage from CCR5 to CXCR4, and induce resistance in R5 HIV-1 that harbors several substitutions in the V3 loop and non-V3 regions. In general, the resistant viruses are able to recognize the CCR5 inhibitor-bound form of CCR5 called as non-competitive resistance [14], [15], [16], [17], [18], [19], [20], [21] if there are no pre-existing X4 variants [22] though a maraviroc-resistant HIV-1 through competitive resistance mechanisms has been reported *in vivo* [23]. SDF-1α (a natural ligand for CXCR4) and CXCR4 inhibitors such as AMD3100 and T134 also induce selection of inhibitor-resistant variants among X4 viruses without changing coreceptor usage [24], [25], [26], [27], [28], [29]. Although these resistant variants contain various mutations in multiple regions of gp120, the majority of mutations accumulate in the V3 loop, and some of these mutations are shared in different resistant variants. These observations indicate that the V3 loop is a crucial region for the acquisition of CXCR4-inhibitor resistance. Thus, the V3 loop is also the principal determinant for resistance to natural ligands and coreceptor inhibitors. Conversely, we have previously induced reversion of HIV-1 from dual-X4 to dual-R5 [30] using the CXCR4 inhibitor T140. The reversion is indeed associated with substitution in the 11th position of the V3 loop from arginine to serine [30], which is consistent with the 11/25th rule. Nevertheless, it remains elusive how coreceptor inhibitors induce evolution of HIV-1 to use different coreceptors or acquire resistance. Here, we selected AMD3100-escape variants from a dual-X4 HIV-1 carrying the V3 loop from CRF01_AE, which has no positively charged amino acids at the 11th or 25th positions and lacks an N-linked glycan in the V3 loop, to elucidate HIV-1 evolution for escape from CXCR4 inhibitors.

## Materials and Methods

### Ethics statement

The study protocol was approved as a part of “the study of immunological and virological analysis in HIV-1 infection (#540) ” by the ethics committee for epidemiology and general study in the Faculty of Life Sciences in Kumamoto University and the National Center for Global Health and Medicine. Written informed consent was obtained from all studied individuals according to the Declaration of Helsinki.

### Reagents and cells

The CXCR4 antagonist AMD3100 [29], [31], CCR5 antagonist MVC [32], and recombinant human sCD4 were supplied by the AIDS Research and Reference Reagent Program, Division of AIDS, National Institute of Allergy and Infectious Diseases (Bethesda, MD, USA). Another CXCR4 inhibitor, T134, was kindly provided by Dr. Hirokazu Tamamura, Tokyo Medical and Dental University, Tokyo, Japan.

The TZM-bl cell line [33] was provided by Dr. John C. Kappes, Dr. Xiaoyun Wu, and Tranzyme through the AIDS Research and Reference Reagent Program, Division of AIDS, National Institute of Allergy and Infectious Diseases, and maintained in Dulbecco's modified Eagle's medium (DMEM) (Sigma) supplemented with 10% fetal bovine serum (FBS) (BioWhittaker). The human embryonic kidney 293T cell line was obtained from American Type Culture Collection (ATCC), and maintained in DMEM supplemented with 10% FBS, 100 U/mL penicillin, and 100 µg/mL streptomycin. The human CD4+ T cell line SupT1 was obtained from ATCC, and its derivative cell line SupT1/CCR5, which expressed high levels of CCR5, was established using a retroviral vector as described previously [13], [21], and maintained in RPMI 1640 (Sigma) medium supplemented with 10% FBS, 0.2 mg/mL G418, 100 U/mL penicillin, and 100 µg/mL streptomycin. The CD4-expressing glioma cell line (NP2/CD4) [34], [35] was provided by Dr. Hoshino (Gunma University), and its derivative cell lines, NP2/CD4/CXCR4, NP2/CD4/CCR5, and NP2/CD4/CXCR4/CCR5, were established as described previously [13] and maintained in Eagle's minimum essential medium (Sigma) supplemented with 10% FBS and appropriate antibiotics.

### Construction of an Env expression vector and infectious molecular clone carrying the V3 loop from CRF01_AE HIV-1

cDNAs of viral RNA from CRF01_AE-infected individuals were prepared as previously described [36]. The *env* region was first amplified using the following primers: 5′-GGTAGAGCAGATGCAGGATG-3′ and 5′-GTGGGTGCTATTCCTAGTGGTTC-3′. Nested PCR was performed using primers carrying *Afl*II and *Nhe*I restriction enzyme sites: 5′-GCACCTTAAGAAATCTGTAGAAATCAATTG-3′ and 5′-GCTAGCTACCTGTTTTAAAGCTTTATACC-3′ (underlines denote *Afl*II and *Nhe*I sites, respectively). The amplified product was then cloned into a pCR-TOPO vector (Invitrogen) and sequenced using an ABI PRISM 3771 automated sequencer (Applied Biosystems). For construction of the Env expression vector carrying the V3 loop from CRF01_AE, the *Afl*II-*Nhe*I fragment of cloned V3 regions was introduced into the *Afl*II-*Nhe*I cloning site of pCXN-JR-FLan [19], [20], [21]. To construct the infectious molecular clone, the *Afl*II-*Nhe*I fragment was similarly introduced into pJR-FLan [19], [20], [21] as described previously, resulting in pJR-FLan carrying the V3 loop from CRF01_AE.

### Construction of infectious molecular clones and Env expression vectors with mutations

Env expression vectors with single or multiple mutations were constructed using *Dra*III, *Eco*RV, *Nhe*I, and *Bsa*BI sites in KI812.7 *env*. Each PCR fragment carrying a mutation was substituted with wild-type *env*, resulting in an Env expression vector with a single mutation. Env expression vectors with different combinations of mutations were constructed by swapping the restriction fragments. Similarly, infectious molecular clones with mutations were constructed as described previously [19], [20], [21].

### Virus preparation

A pseudotyped virus carrying the luciferase gene was prepared as previously described [13]. Briefly, 293T (3×10^6^ cells) were transfected with 20 µg pNL-LucΔBglII and 10 µg pCXN-Env vectors. To produce infectious viral clones, 293T cells were transfected with 30 µg infectious HIV-1 clones. Virus-containing culture supernatants were recovered at 48 h post-transfection, filtered through a 0.22-µm filter (Millipore), and then stored at −80°C until use. The p24 Gag in the supernatant was measured using a p24 Ag ELISA (Zeptometrix) according to the manufacturer's protocol.

### Isolation of AMD3100-escape variants from HIV-1_JR-FLan/KI812.7_

To isolate AMD3100-escape variants from HIV-1_JR-FLan/KI812.7_, the virus was passaged in SupT1/CCR5 cells with increasing concentrations of AMD3100. Viral replication was monitored by observing the cytopathic effect on SupT1/CCR5 cells. After 21 passages of the virus in SupT1/CCR5 cells at a final AMD3100 concentration of 4 µM, AMD3100 was removed from the virus-infected cell cultures, and the virus was recovered from the culture supernatant. The sensitivity of the escape variant to coreceptor antagonists was determined using TZM-bl cells. DNA was extracted from virus-infected cells using a QIAamp DNA Blood kit (Qiagen) and then subjected to PCR using *Taq* DNA polymerase (Promega). The V3 region sequences were amplified using the following primers: 5′-GCACCTTAAGAAATCTGTAGAAATCAATTG-3′ and 5′-GCTAGCTACCTGTTTTAAAGCTTTATACC-3′. The amplified products were cloned into pCR-TOPO (Invitrogen), and then the *env* regions of the virus were sequenced using the ABI PRISM 3130 automated sequencer.

### Determination of drug sensitivity of replication-competent viruses

The sensitivity of replication-competent viruses to coreceptor inhibitors was determined using TZM-bl or SupT1/CCR5 cells. For TZM-bl cells, the cells were infected with viruses at 37°C for 2 days in the presence of various concentrations of coreceptor inhibitors. Luciferase activities of the cells were measured using a luminometer (Lumat LB 9501/16; Berthold). The sensitivity of the virus to coreceptor inhibitors was expressed as the 50% effective concentration (EC_50_), which was the drug concentration that reduced infection levels by 50% compared with that in the infected, drug-free control of triplicate experiments. For SupT1/CCR5 cells, 5×10^3^ cells in U-bottom 96-well microplates were infected with the same amount of virus (100 TCID_50_) in the presence of various AMD3100 concentrations, and then cultured for 6 days. The cytopathic effect was determined using an MTT assay as described previously [37].

### Determination of drug sensitivity and coreceptor usage of pseudotyped viruses

To determine the coreceptor inhibitor sensitivity of pseudotyped viruses carrying the luciferase gene, NP2/CD4 cells expressing both CCR5 and CXCR4 were used as target cells. Briefly, the target cells (1.5×10^4^ cells) were seeded in 48-well culture plates. The following day, the cells were incubated in the presence or absence of various concentrations of coreceptor inhibitors at 37°C for 30 min. The virus (50 ng p24 Ag) was then added to the cells and incubated at 37°C for 48 h. Luciferase activities of the cells were measured using the luminometer. The sensitivity of the virus to coreceptor inhibitors was expressed as the EC_50_. To examine the coreceptor usage of the virus, NP2/CD4 cells expressing either CCR5 or CXCR4 were infected with pseudotyped viruses carrying the luciferase gene. Luciferase activities were measured after 48 h of infection in triplicate experiments using the luminometer.

### Determination of entry efficiency of the virus

Entry efficiency of the virus was determined using a single-round replication assay. Briefly, NP2/CD4/CXCR4/CCR5 cells were infected with the same amount (10 ng p24 Ag) of pseudotyped HIV-1 carrying the luciferase gene. Luciferase activity was measured at 48 h post-infection using the luminometer.

## Results

### Coreceptor usage of a CRF01_AE-derived HIV-1 and its sensitivity to coreceptor inhibitors

**Figure 1:**
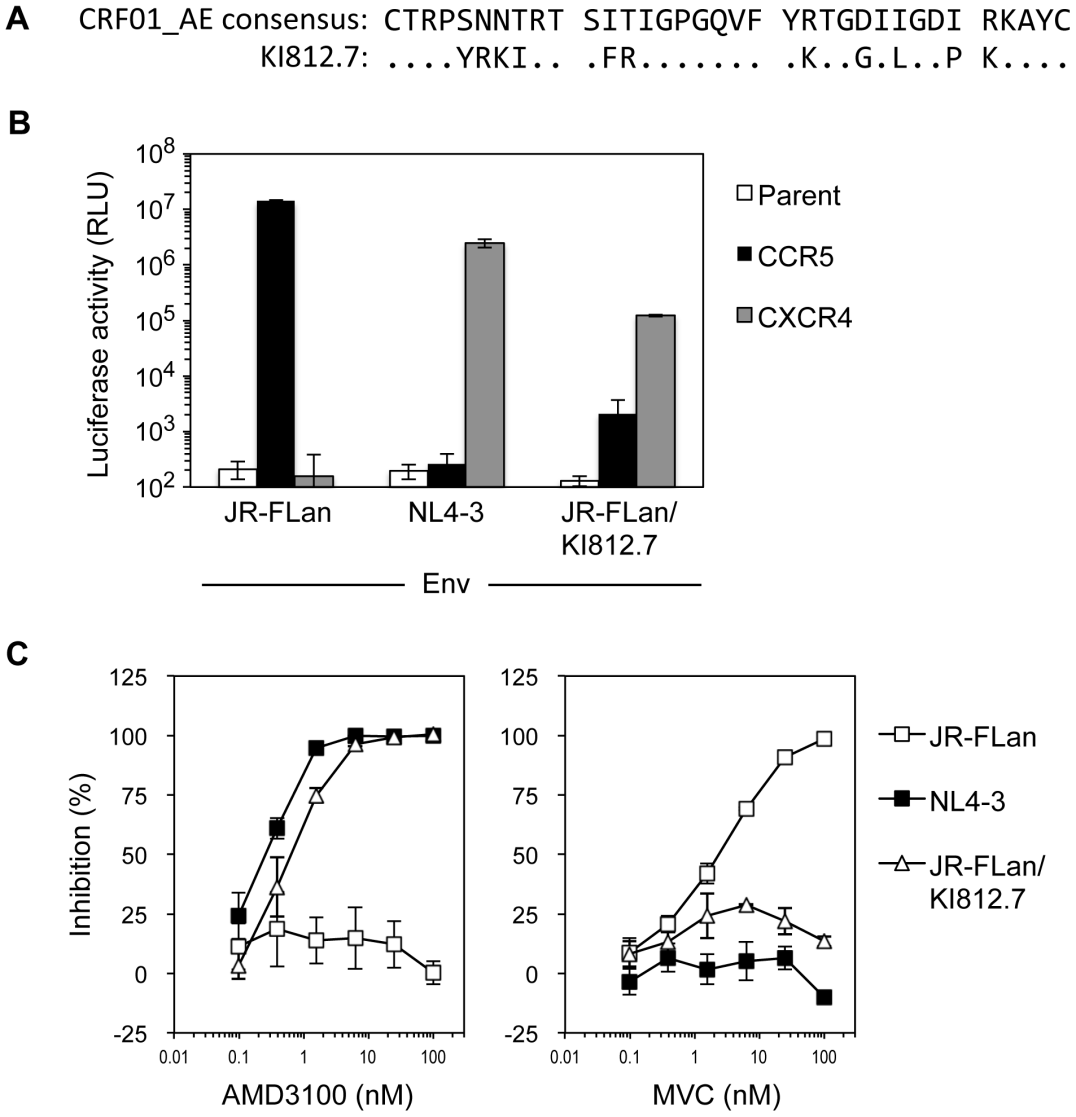
Virological characterization of HIV-1 carrying the V3 loop from CRF01_AE KI812.7. (A) V3 loop amino acid sequences of CRF01_AE consensus and KI812.7. Dots denote sequence identity. (B) Coreceptor usage of JR-FLan carrying the V3 loop from KI812.7. NP2/CD4 cells and NP2/CD4 cells expressing CCR5 or CXCR4 were infected with the same amount of luciferase-reporter pseudotyped virus (10 ng p24Ag). Luciferase activities were measured at 48 h post-infection. Data are the geometric means ± SD of triplicate experiments. (C) Susceptibility of replication-competent HIV-1_JR-FLan/KI812.7_. TZM-bl cells were infected with replication-competent virus in the presence of AMD3100 or maraviroc (MVC), and then the luciferase activities of the infected cells were measured at 48 h post-infection. Data represent the extent of inhibition of replication relative to that in the absence of AMD3100 or MVC.

We previously isolated a CXCR4 inhibitor-escape variant from dual-X4 HIV-1 89.6, which has a substitution at the 11th position of the V3 loop [30]. This change does not confer reduced sensitivity to CXCR4 inhibitors, but induces reversion of dual-X4 to dual-R5. However, it remains to be determined how CXCR4-using HIV-1 without a positively charged amino acid at the 11th position of the V3 loop escapes from CXCR4 inhibitors. Since higher prevalence of CXCR4-using HIV-1 in CRF01_AE compared to subtype B has been reported [38], we first cloned and sequenced the *env* regions of HIV-1s from 21 CRF01_AE-infected individuals in a Japanese cohort to find CXCR4-using HIV-1 lacking positively charged amino acids at the 11th and 25th positions of the V3 loop. Among them, two out of five clones isolated from individual KI812 had a unique amino acid sequence (KI812.7) as shown in Fig. 1A. Although the 11th and 25th positions of the V3 loop did not contain charged amino acids, the net charge of the V3 loop was +7. Furthermore, there was no putative N-linked glycosylation site at the 6th position. Geno2pheno coreceptor algorithms [39] (http://coreceptor.bioinf.mpi-inf.mpg.de/) predicted that the virus was capable of using CXCR4 as a coreceptor (false positive rate: 0.1%). To confirm the coreceptor usage of the virus, an Env expression vector and an infectious molecular clone carrying the V3 loop derived from KI812.7 were constructed using pJR-FL as a backbone, which were designated as pCXN-FLan/KI812.7 and pJR-FLan/KI812.7, respectively. As we reported previously, the virus pseudotyped with JR-FLan and NL4-3 Env exclusively infected NP2/CD4 cells expressing CCR5 and CXCR4, respectively (Fig. 1B). In contrast, luciferase activity of CXCR4-expressing cells infected with virus carrying FLan/KI812.7 Env was ∼100-fold higher than that of CCR5-expressing cells, indicating that FLan/KI812.7 Env preferentially used CXCR4 over CCR5. These results confirmed that substitution of the V3 loop with KI812.7 changed coreceptor usage from R5 to X4 (Fig. 1B). Furthermore, an infectious clone, HIV-1_JR-FLan/KI812.7_, was sensitive to the CXCR4 inhibitor AMD3100 (EC_50_ value: 0.62±0.21 nM) as well as X4 HIV NL4-3 (EC_50_ value: 0.26±0.04 nM), but resistant to the CCR5 inhibitor MVC in both CCR5- and CXCR4-expressing TZM-bl cells (Fig. 1C). Taken together, the virus carrying JR-FLan/KI812.7 Env was a dual-X4 HIV-1.

### Selection of AMD3100-resistant variants from HIV-1_JR-FLan/KI812.7_

**Figure 2:**
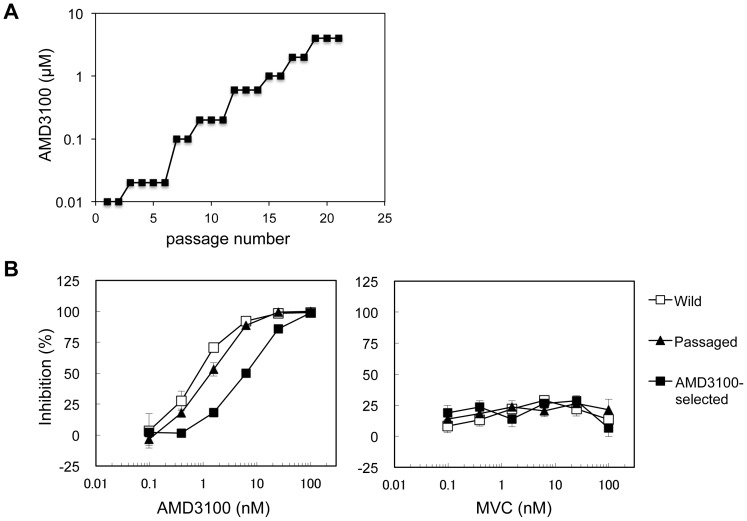
Selection of AMD3100-escape variants from HIV-1_JR-FLan/KI812.7_. (A) Induction of AMD3100-resistant variants from HIV-1_JR-FLan/KI812.7_. Replication-competent HIV-1_JR-FLan/KI812.7_ was passaged using SupT1/CCR5 cells in increasing concentrations of AMD3100 in the range of 20 nM to 4 µM. (B) Susceptibilities of AMD3100-selected variants to AMD3100 and MVC. TZM-bl cells were treated with various concentrations of AMD3100 or MVC, and infected with wild-type HIV-1_JR-FLan/KI812.7_, the virus passaged in the absence of AMD3100, or the selected virus in the presence of 4 µM AMD3100. Luciferase activities of TZM-bl cells were measured at 48 h post-infection. Data represent the extent of inhibition of replication relative to that in the absence of AMD3100 or MVC.

To elucidate how CXCR4-using HIV-1 escapes from the CXCR4 inhibitor AMD3100, we isolated AMD3100-escape variants from HIV-1_JR-FLan/KI812.7_ using a SupT1 cell line expressing high levels of CCR5. This cell line was able to support both CXCR4- and CCR5-using HIV-1 replication, thereby permitting both resistance to AMD3100 and coreceptor switching of the virus. To select AMD3100-escape variants, SupT1/CCR5 cells were passaged in increasing concentrations of AMD3100. The virus was also passaged in the absence of AMD3100 to exclude the effect of long-term culture. After 21 passages of the virus in the presence of 4 µM AMD3100 (Fig. 2A), the virus was recovered and its sensitivity to AMD3100 was determined using TZM-bl cells. As a result, the selected virus displayed reduced sensitivity (4-fold) to AMD3100 compared with that of the passaged virus in the absence of AMD3100 and the wild-type virus (Fig 2B). The EC_50_ value of the selected virus was 62 nM, whereas that of the passaged virus was 14 nM. Furthermore, entry of the selected virus was completely inhibited by high concentrations of AMD3100, and the virus was completely resistant to MVC in TZM-bl cells. These results suggested an absence of coreceptors switching from CXCR4 to CCR5 and a competitive resistance profile of the virus to AMD3100.

### Amino acid sequences of the AMD3100-resistant HIV-1

**Figure 3:**
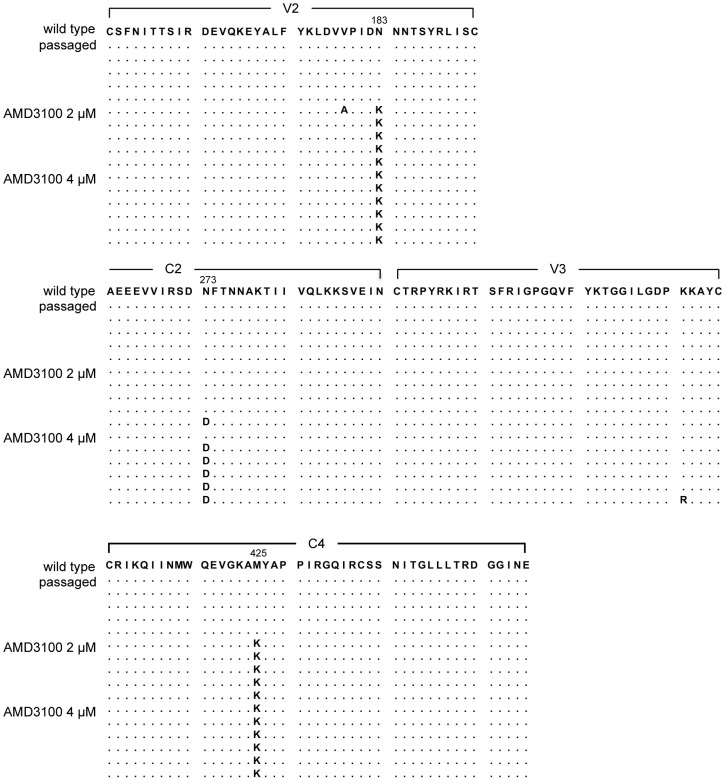
Amino acid sequences of the AMD3100-escape variant. Amplified products from infected SupT1/CCR5 cells in the absence or presence of AMD3100 were cloned, and five to six clones from each sample were sequenced. The amino acid sequences of V2, C2, and C4 of the wild-type HIV-1_JR-FLan/KI812.7_ are shown in the top line. In each set of clones, the deduced amino acid sequence was aligned by the single amino acid code. Identity with this sequence at individual amino acid positions is indicated by dots.

To determine which regions were responsible for the reduced sensitivity of the escape variant to AMD3100, the V1–C4 regions of the envelope gene were sequenced using DNA amplified from infected cells as a template. In the selected virus at 2 µM AMD3100, the virus harbored an N138K substitution in the V2 region and a M425K substitution in the C4 region. Furthermore, the escape variant acquired an N273D substitution in the C2 region at 4 µM AMD3100 (Fig. 3). Most clones passaged in the presence of AMD3100 did not have substitutions in the V3 loop (one clone had a K to R substitution at the 31th position of the V3 loop). In contrast, no remarkable changes were observed in the passaged virus in the absence of AMD3100 (Fig. 3).

### Non-V3 regions are involved in the reduced sensitivity to AMD3100

**Figure 4:**
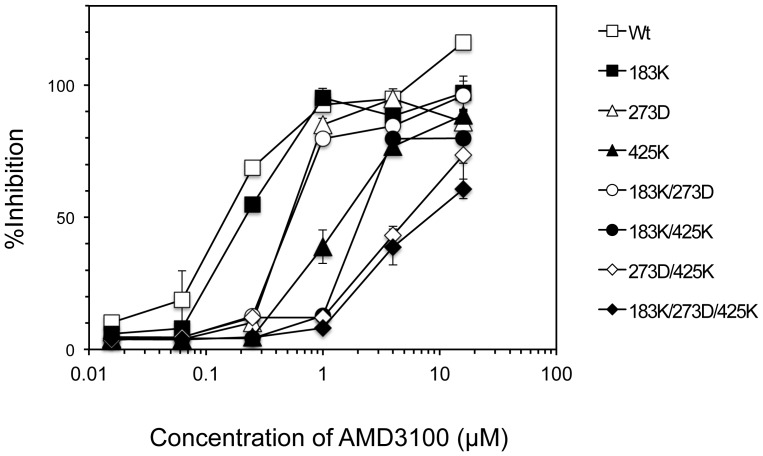
Susceptibilities of replication-competent recombinant viruses. SupT1/CCR5 cells were infected with the same amount of replication-competent recombinant viruses carrying mutations (100 TCID_50_) in the presence of various concentrations of AMD3100, and then cultured for 6 days. Cytopathic effects were determined by an MTT assay. Data are the means ± SD of triplicate experiments.

To examine which substitutions were responsible for the reduced sensitivity to AMD3100, we constructed and produced infectious molecular clones carrying single or multiple mutations. The sensitivity of each mutant was then determined by an MTT assay using SupT1/CCR5 cells (Fig. 4, Table 1). In single mutants, M425K and N273D conferred 10-fold and 3-fold reduced sensitivities to AMD3100, respectively, whereas N183K was almost dispensable. Furthermore, mutants carrying both N273D and M425K (N273D/M425 and N183K/N273D/M425K) conferred a more than 40-fold reduced sensitivity to AMD3100. To confirm the sensitivity of mutants to AMD3100 using a single-round entry assay, we constructed Env expression vectors carrying single or multiple mutations. Pseudotyped viruses carrying the luciferase gene were produced by cotransfection of 293T cells with these vectors and an *env*-lacking luciferase-reporter HIV-1 construct. The sensitivity of each mutant was then determined using NP2/CD4 cells expressing both CXCR4 and CCR5 (Table 2). In single mutants, N273D and M425K substitutions conferred reduced sensitivity to AMD3100 (4.1-fold and 2.6-fold, respectively), whereas N183K had a minor effect (1.5-fold) as shown in Table 2. The N293D mutation combined with M425K (273D/425K) conferred increased resistance to AMD3100 (10-fold). In contrast, addition of N183K had a minor effect on the reduced sensitivity to AMD3100 in combination with N273D/M425K (13-fold). These results indicated that both N273D and M425K were mainly involved in the reduced sensitivity to AMD3100. The reduced sensitivity to AMD3100 was thus independent of the V3 loop.

**Table 1:** Susceptibility of recombinant viruses to AMD3100 determined by MTT assays.

virus	EC_50_ (µM)^a^
	AMD3100
wild type	0.15±0.02^b^(1.0)
183K	0.21±0.01 (1.5)
273D	0.52±0.02 (3.6)
425K	1.5±0.23(10)
183K/273D	0.54±0.01 (3.7)
183K/425K	2.2±0.05(14)
273D/425K	5.9±1.4(40)
183K/273D/425K	10.3±2.5(70)

a SupT1/CCR5 cells (5×10^3^) were infected with 100TCID_50_ recombinant viruses, and then the cytotoxicity induced by HIV-1 was measured at day 6 post-infection by an MTT assay to determine the effective concentration of 50% inhibition (EC_50_).

b Mean ± SD (n = 3). Numbers in parenthesis represent fold changes of EC_50_ values compared with that of the wild type.

**Table 2:** Susceptibilities of recombinant pseudotyped viruses to CXCR4 inhibitors determined by single-round entry assays.

virus	EC_50_ (µM)^a^			
	AMD3100		T134	
wild type	0.012±0.0047^b^	(1.0)	0.033±0.0086	(1.0)
183K	0.018±0.0015	(1.5)	0.023±0.0064	(0.7)
273D	0.052±0.029	(4.1)	0.034±0.0010	(1.0)
425K	0.032±0.012	(2.6)	0.097±0.011	(2.9)
183K/273D	0.031±0.015	(2.4)	0.054±0.014	(1.6)
183K/425K	0.020±0.012	(1.6)	0.062±0.011	(1.9)
273D/425K	0.12±0.014	(10)	0.061±0.013	(1.8)
183K/273D/425K	0.16±0.031	(13)	0.099±0.013	(3.0)

a NP2/CD4/CXCR4/CCR5 cells (1.5×10^4^) were infected with pseudotyped virus (50 ng p24Ag) in the presence of CXCR4 inhibitors, and then the luciferase activity was measured at 48 h post-infection to determine the effective concentration of 50% entry inhibition (EC_50_).

b Mean ± SD (n = 3). Numbers in parenthesis represent fold changes of EC_50_ values compared with that of the wild type.

We next determined whether viruses carrying these mutations were cross-resistant to another CXCR4 inhibitor, T134 [24] (Table 2). We found that a single M425K mutation and combinations with M425K were cross-resistant to T134 (3-fold). However, similar to the wild-type, N273D was sensitive to T134.

### Involvement of the C4 region in enhanced replication of AMD3100-resistant HIV-1

**Figure 5:**
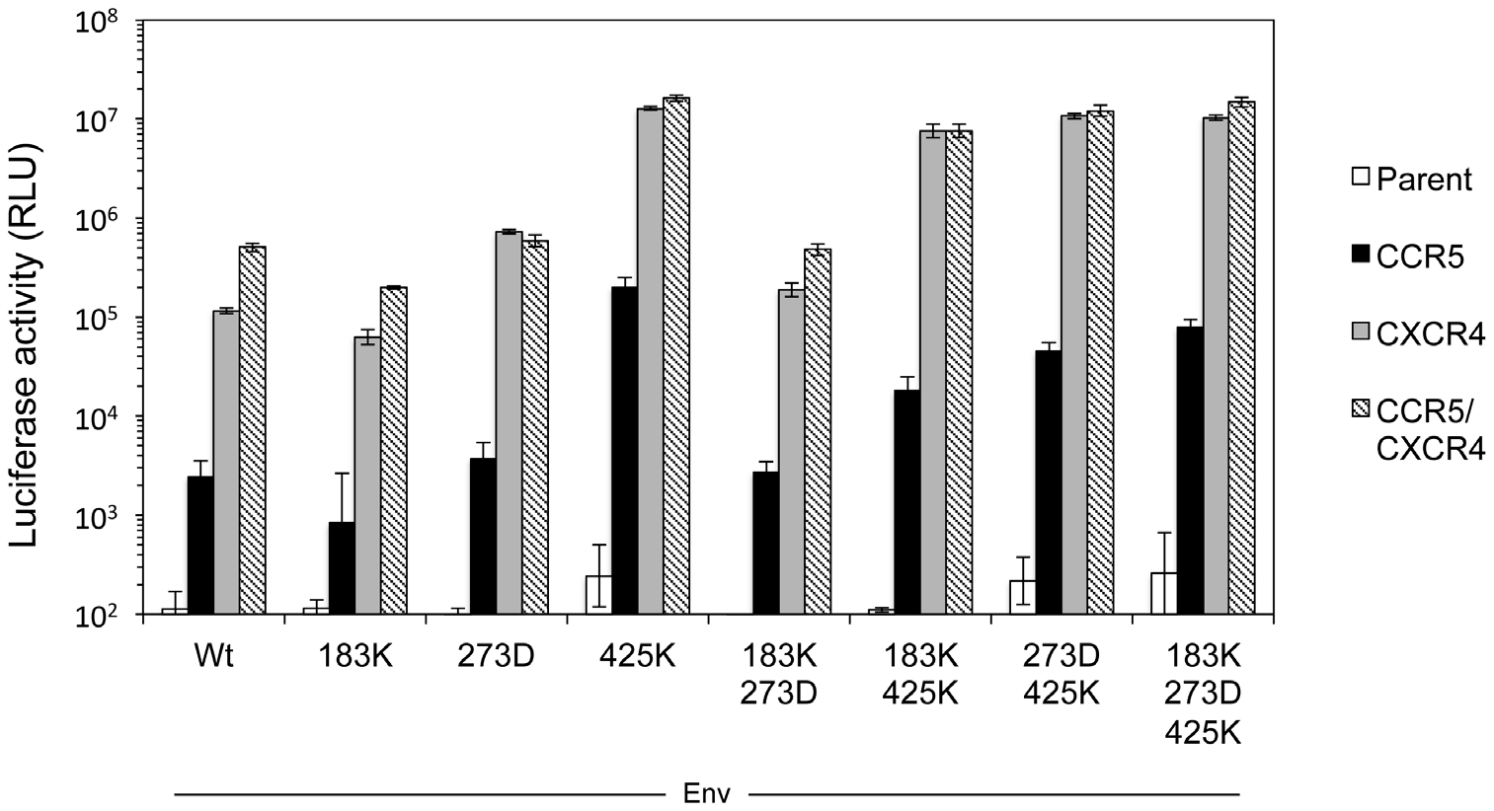
Coreceptor usage and entry efficiency of recombinant pseudotyped HIV. Coreceptor usage of the recombinant luciferase-reporter HIV was determined using NP2/CD4 cells expressing either CCR5 or CXCR4. Entry efficiencies of recombinant pseudotyped HIVs were determined using NP2/CD4 cells expressing both CXCR4 and CCR5. The cells were infected with the same amount of luciferase-reporter pseudotyped virus (10 ng p24Ag) with the indicated mutations. Luciferase activities were measured at 48 h post-infection. Data are the geometric means ± SD of triplicate experiments.

**Figure 6:**
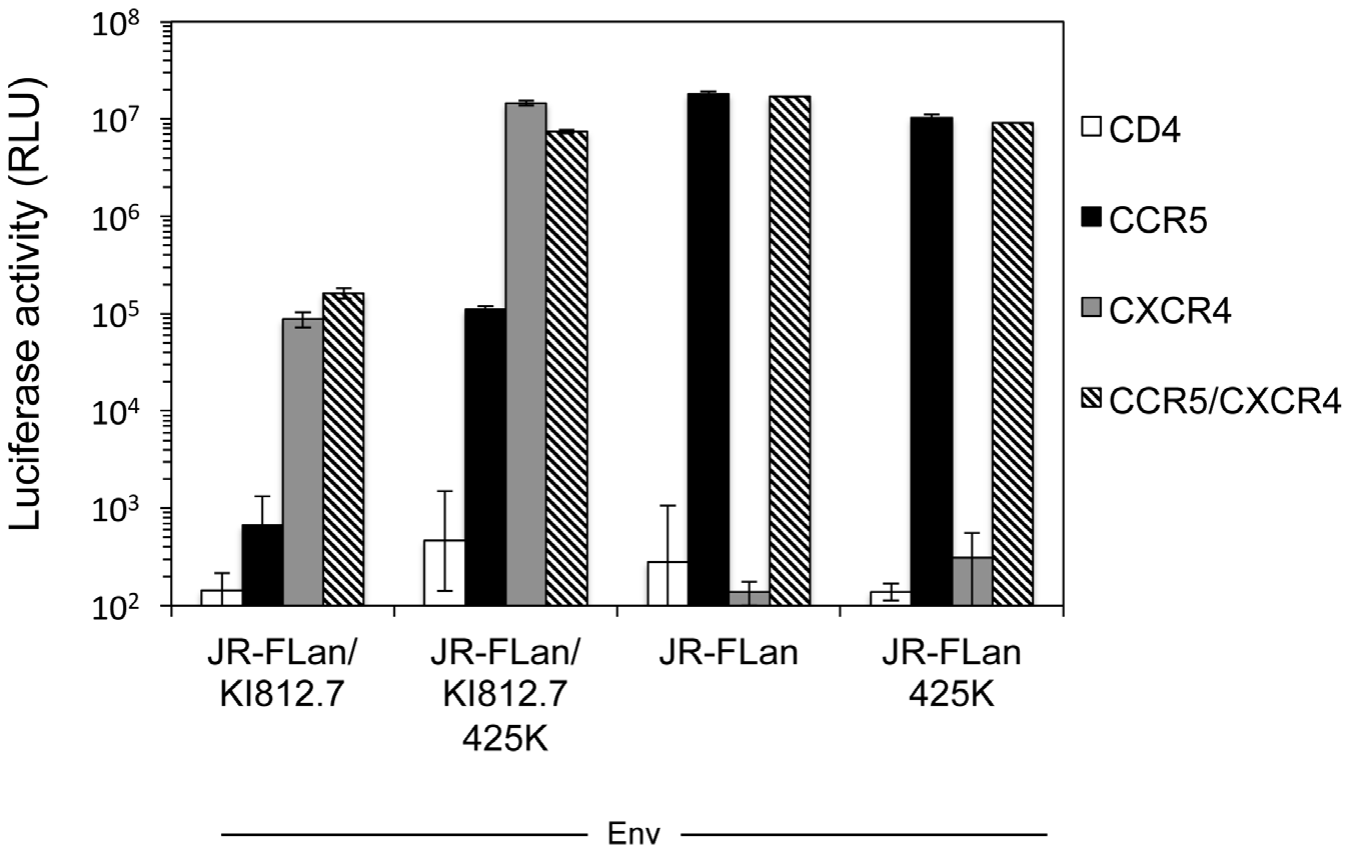
Effect of M425K substitution on JR-FLan Env. Coreceptor usage and entry efficiency of recombinant luciferase-reporter HIV pseudotyped with the indicated Envs were determined using NP2/CD4 cells expressing either CXCR4 or CCR5 and cells expressing both CXCR4 and CCR5, respectively. The cells were infected with the same amount of luciferase-reporter pseudotyped virus (10 ng p24Ag) with the indicated mutations. Luciferase activities were measured at 48 h post-infection. Data are the geometric means ± SD of triplicate experiments.

We next evaluated whether these mutations changed the coreceptor preference from CXCR4 to CCR5. To this end, NP2/CD4 cells expressing either CCR5 or CXCR4 were infected with the luciferase-reporter HIV-1 pseudotyped with single or multiple mutations (Fig. 5). After infection of CXCR4-expressing cells with all recombinant viruses derived from JR-FLan/KI812.7, luciferase activities were ∼100-fold higher than those of CCR5-expressing cells. This result indicated that all Envs, including N183K, N273D, and M425K, did not change preferential use of CXCR4. We also determined the entry efficiencies of the mutants using NP2/CD4 cells expressing both CXCR4 and CCR5. Luciferase activities of the cells infected with the same amount of the viruses (10 ng p24 Ag) showed that the single M425K substitution, but not N183K and N273D, increased the entry efficiency compared with that of the wild-type virus (Fig. 5). Mutations combined with 425K (183K/425K, 273/425K, and 183K/273D/425K) also had similar infectivities to that of the single mutation (Fig. 5), indicating that M425K substitution was essential for enhancement of viral infectivity. We next determined whether the M425K substitution also enhanced the entry efficiency of JR-FLan Env. However, M425K substitution in JR-FLan Env neither increased the luciferase activity in NP2/CD4/CCR5/CXCR4 (Fig. 6) nor changed coreceptor usage from CXCR4 to CCR5. These results indicated that the enhanced infectivity by M425K substitution was only observed in the context of Env carrying the V3 loop from KI812.7.

### Involvement of C2 and C4 regions in the increased sensitivity to soluble CD4

Because the change in the C2 region (N273D) was located in loop D, which is associated with resistance to the monoclonal neutralizing antibody VRC01 and soluble (s)CD4 [40], we examined whether the mutations also affected the sensitivity to sCD4 (Table 3). We found that the wild-type virus was resistant to sCD4 (the EC_50_ value was more than 10 µg/ml). N183K mutation did not change the sensitivity, whereas N273D increased the sensitivity to sCD4 to some extent (EC_50_: 6.9±0.26 µg/mL). In contrast, M425K largely increased the sensitivity to sCD4 (EC_50_: 0.42±0.04 µg/mL). These results indicated that not only the C2 mutation but also the C4 region mutation affected the increased sensitivity to sCD4.

**Table 3:** Susceptibility of recombinant pseudotyped viruses to sCD4 determined by single-round entry assays.

virus	EC_50_ (µg/mL)^a^
	sCD4
wild type	>10
183K	>10
273D	6.9±0.26^b^
425K	0.42±0.040
183K/273D	8.6±0.89
183K/425K	0.35±0.024
273D/425K	0.14±0.0033
183K/273D/425K	0.22±0.016

a NP2/CD4/CXCR4/CCR5 cells (1.5×10^4^) were infected with pseudotyped virus (50 ng p24Ag), and then the luciferase activity was measured at 48 h post-infection to determine the effective concentration of 50% entry inhibition (EC_50_).

b Mean ± SD (n = 3).

## Discussion

Characterization of CXCR4 inhibitor-resistant HIV-1 is important to understand how the virus can escape from inhibitors targeting coreceptors although clinical application of CXCR4 inhibitors for treatment of HIV-1-infected individuals remains a matter of debate. In the present study, we successfully isolated an AMD3100-escape variant from dual-X4 HIV-1. Interestingly, the variants had substitutions in C2 and C4 regions (N273D and M425K, respectively), which were responsible for their resistance to AMD3100 based on site-directed mutagenesis experiments. In contrast, no remarkable changes were observed in the V3 loop. In general, the V3 loop is a crucial determinant for coreceptor selectivity and resistance to coreceptor inhibitors and natural ligands. CXCR4 inhibitor-resistant X4 viruses show numerous mutations in the V3 loop and other regions, although the responsible region(s) have mostly not been determined for the reduced sensitivity to CXCR4 inhibitors [24], [25], [26], [27], [28]. In our previous study, we also selected a CXCR4 inhibitor-escape variant from dual-X4 HIV-1, which had serine to arginine substitution at the 11th position of the V3 loop [30]. *In vitro* experiments have revealed that coreceptor selectivity of HIV-1 is determined by the amino acid sequence of gp120, particularly the number [41], [42] and position of charged amino acids in the V3 loop such as the 11/25 rule. Thus, amino acid substitution in the V3 loop can predict the loss of CXCR4 usage. Indeed, mutational analysis confirmed reversion of dual-X4 to dual-R5 by substitution. Conversely, the dual-X4 virus used in this study did not have a positively charged amino acid at the 11th or 25th position of the V3 loop, such as arginine or lysine. However, the Geno2pheno coreceptor algorithm predicted CXCR4 use of this virus because of an increased net positive charge and lack of an N-linked glycan in the V3 loop. In fact, analyses of coreceptor usage revealed that the virus carrying the V3 loop from KI812.7 predominantly used CXCR4 as the coreceptor. Furthermore, the AMD3100-escape variant was found to predominantly use CXCR4 without reversion from CXCR4 to CCR5. Therefore, it is likely that viruses not carrying a charged amino acid at the 11th position of the V3 loop lose their ability to revert from CXCR4 to CCR5 use. To acquire resistance to CXCR4 inhibitors, such viruses may need to induce substitutions in the V3 loop or different regions of gp120, such as C2 and C4 regions.

It has been suggested that CXCR4 inhibitor-resistant viruses exhibit reduced fitness [25], probably because of lower affinity of gp120 for CXCR4. Notably, M425 is located in the β21 sheet of the gp120 bridging sheet that is thought to be important for coreceptor binding together with the stem of the V3 loop (V3 stem) [43], [44], [45]. Because there was no reduction in the maximum plateau inhibition [18] for AMD3100 in this escape variant, it is unlikely that the selected virus recognized the AMD3100-bound form of CXCR4 [46]. Instead, sufficient concentrations of AMD3100 reached∼100% inhibition of the selected virus (right shift in the EC_50_ value), indicating competitive resistance. It is thus possible that the M425K substitution may alter the binding affinity for CXCR4 in the context of the V3 loop from KI812.7 [47], [48], [49] to retain the viral replication fitness. Indeed, the M425K substitution was cross-resistant to another CXCR4 inhibitor, T134, and dramatically enhanced the entry efficiency of the virus carrying the V3 loop from KI812.7 (∼100-fold) but not from the JR-FLan background. Other studies have also shown that escape from the CCR5 inhibitor vicriviroc is not associated with a loss of fitness [50], which is not caused by changes in the V3 loop, similar to our variant, but rather substitutions in the fusion peptide domain of gp41 [51]. Taken together, it is possible that coreceptor inhibitor-resistant viruses need to retain or increase the affinity for their coreceptors by changing V3 or non-V3 regions, which are probably dependent on the configuration of the V3 loop.

In contrast, the N273D substitution was also shown to be an important determinant for reduced sensitivity to AMD3100, although the mutation did not significantly increase the entry efficiency of the virus. In fact, N273D is located in loop D of the C2 region and is associated with the loss of N-glycan, indicating alteration of the whole structure of gp120 via steric hindrance. It has been shown that N273A affects sensitivities to the broadly neutralizing monoclonal antibody VRC01 and sCD4 [40]. The structure of VRC01 in complex with the gp120 core revealed that the VRC01 heavy chain binds to the gp120 CD4bs in a manner similar to that of CD4 [52]. Indeed, our mutations, not only N273D but also M425K, conferred sensitivity to sCD4, suggesting that these substitutions affect the CD4 binding affinity. It has been reported that AMD3100 directly interacts with Asp^171^ and Asp^262^ of CXCR4 [53], as well as ECL2 and TM4 [54]. However, inhibitory activities of AMD3100 in CXCR4 mutants at these positions are dependent on the strain of CXCR4-using HIV-1 [53]. Thus, different CXCR4-using HIV-1s vary in their dependence on residues in one or the other domains [55]. Taken together, it is possible that gp120 with N273D or M425K might recognize a different portion of the CD4/CXCR4 complex and alter their affinity. However, structural analysis of the gp120 core carrying these mutations with the V3 loop from KI812.7 is necessary to address these issues.

In conclusion, it is possible to induce a CXCR4 inhibitor-resistant virus from CXCR4-using HIV-1 without changing the V3 loop. The configuration of the V3 loop might be the major determinant for selection of such resistant viruses, which may also determine how the virus evolves for resistance or the coreceptor switch. Further structure-based analyses are necessary to elucidate these molecular mechanisms.
